# Therapeutic effect of adipose stromal vascular fraction spheroids for partial bladder outlet obstruction induced underactive bladder

**DOI:** 10.1186/s13287-022-02739-w

**Published:** 2022-02-09

**Authors:** Jingyu Liu, Liuhua Zhou, Feng Zhao, Changcheng Zhou, Tianli Yang, Zhongle Xu, Xinning Wang, Luwei Xu, Zheng Xu, Yuzheng Ge, Ran Wu, Ruipeng Jia

**Affiliations:** 1 Department of Urology, Nanjing First Hospital, Nanjing Medical University, No. 68 Changle Road, Nanjing, 210006 Jiangsu People’s Republic of China; 2 Department of Urology, Hefei Hospital Affiliated to Anhui Medical University (The Second People’s Hospital of Hefei), Hefei, Anhui People’s Republic of China; 3 Department of Urology, The Affiliated Hospital of Qingdao University, Qingdao, Shandong People’s Republic of China

**Keywords:** Stromal vascular fraction, Spheroid, Bladder outlet obstruction, Underactive bladder

## Abstract

**Background:**

Underactive bladder (UAB) is a common clinical problem but related research is rarely explored. As there are currently no effective therapies, the administration of adipose stromal vascular fraction (ad-SVF) provides a new potential method to treat underactive bladder.

**Methods:**

Male Sprague–Dawley rats were induced by partial bladder outlet obstruction (PBOO) for four weeks and randomly divided into three groups: rats treated with PBS (Sham group); rats administrated with ad-SVF (ad-SVF group) and rats performed with ad-SVF spheroids (ad-SVFsp group). After four weeks, urodynamic studies were performed to evaluate bladder functions and all rats were sacrificed for further studies.

**Results:**

We observed that the bladder functions and symptoms of UAB were significantly improved in the ad-SVFsp group than that in the Sham group and ad-SVF group. Meanwhile, our data showed that ad-SVF spheroids could remarkably promote angiogenesis, suppress cell apoptosis and stimulate cell proliferation in bladder tissue than that in the other two groups. Moreover, ad-SVF spheroids increased the expression levels of bFGF, HGF and VEGF-A than ad-SVF. IVIS Spectrum small-animal in vivo imaging system revealed that ad-SVF spheroids could increase the retention rate of transplanted cells in bladder tissue.

**Conclusions:**

Ad-SVF spheroids improved functions and symptoms of bladder induced by PBOO, which contributes to promote angiogenesis, suppress cell apoptosis and stimulate cell proliferation. Ad-SVF spheroids provide a potential treatment for the future patients with UAB.

## Background

Underactive bladder (UAB) is a chronic and complicated urinary system disease related to detrusor underactivity (DUA), which is a common clinical problem for patients with lower urinary tract symptoms (LUTS) [1]. UAB also can lead to severe voiding and storage disorder, such as urinary retention, recurrent urinary tract infections, and even kidney damage. In 2002, the International Continence Society (ICS) defined the urodynamic diagnosis of DUA as “a contraction of reduced strength and/or duration, resulting in prolonged bladder emptying and/or a failure to achieve complete bladder emptying within a normal time span” [2]. Previous study suggested that the prevalence of DUA among young men (aged < 50 years) is 9% to 28%, while it rises to 48% among older men (aged > 70 years) [3]. Additionally, the prevalence of DUA is about 12% to 45% in elderly women [4].

Partial bladder outlet obstruction (PBOO) is a major myogenic disease that leads to UAB and usually causes by benign prostatic hyperplasia (BPH) or pathologies narrowing the outlet, such as urethral stricture or bladder neck contracture [5]. Currently, the mechanism between PBOO and UAB is not clear. PBOO may change the ultrastructure of the detrusor muscle and its gap junctions, then resulting in the dysfunction of ion storage, transport, and energy metabolism in detrusor muscle [6, 7]. In obstructed bladder, the blood flow reduced due to effects of the increased tissue pressure in bladder wall during filling and/or the raised intravesical pressure during voiding [7]. The above reasons can lead to detrusor muscle ischemia and hypoxia in the bladder, which increase the expression of transforming growth factor-β (TGF-β), Collagen type I, and Collagen type III [8]. However, the symptoms of UAB still exist after the PBOO resolved in part of patients [4]. There is currently no an effective therapeutic approach to UAB.

Up to now, various of treatments were attempted to attenuate symptoms and improve bladder functions of UAB [9–11]. Stem cell therapy has attracted widespread attention as an effective approach to the regeneration of tissues. Adipose-derived regenerative cells have been explored to improve bladder dysfunction and histological changes induced by bladder over-distention [9]. Adipose-derived stromal vascular fraction (ad-SVF) as safe and low-cost stem cell therapy methods may be useful in clinic practice, which can be easily and safely harvested from human adipose in large quantities [12, 13]. Recently, our studies have demonstrated that ad-SVF could attenuate acute renal/testicular ischemia reperfusion injury and improve them functions [14, 15]. However, one major shortcoming of directing injection is the low retention rate of cells [16, 17]. As such, culture-dependent self‐aggregation or other assembly of cells into aggregates has been proposed to reduce cells loss [17, 18]. Studies have reported that spheroids from ad-SVF or mesenchymal stem cells (MSC) could enhance angiogenesis and preserve cardiac functions or promote wound repair [19, 20]. Thus, to our knowledge, there is currently no research on the improvement and recovery of UAB functions with the administration of ad-SVF spheroids.

We hypothesize that ad-SVF spheroids may promote the improvement of functions and symptoms in PBOO-induced UAB. Therefore, the current study has been designed to explore the potential therapeutic effects of ad-SVF spheroids in UAB induced by PBOO in a rat model.

## Materials and methods

### Animals

Male Sprague–Dawley (SD) rats weighing 220–320 g were housed at the Experimental Animal Center of Nanjing First Hospital on a 12-h light/12-h dark cycle and with untrammeled access to food and water. All procedures were permitted by the Ethics Committee for the use of Experimental Animals of Nanjing First Hospital, Nanjing Medical University. This study was directed according to the Guidelines for the Care and Use of Laboratory Animals of the National Institutes of Health (NIH).

### Isolation and characterization of ad-SVF

Adipose tissue around epididymis was collected and ad-SVF were isolated according to our previous protocol [14, 15]. Briefly, adipose tissue was extracted and washed with ice-cold sterile phosphate-buffered saline (PBS) thrice. Then, after being cut into small pieces, the adipose tissue was digested with 0.075% type I collagenase at 37 °C for 30–45 min with continuous shaking. The cell pellet was filtered with 200-μm nylon mesh, collected, centrifuged with 400 g for 5 min, washed with PBS twice. After red blood cells being treated with lysis solution for 5 min, the cell was centrifuged with 400 g for 5 min and resuspended with PBS. At last, isolated cells were counted with an automated cell counter.

Isolated cells were performed to explore cell surface marker expression of isolated ad-SVF by flow cytometric analysis. The following antibodies were examined for cell surface markers: Fluorescein Isothiocyanate (FITC) conjugated anti-CD34 (BioLegend), Phycoerythrin (PE) conjugated anti-CD31 (BioLegend), PE conjugated anti-CD45 (BioLegend), PE conjugated anti-CD106 (BioLegend), PE conjugated anti-CD133 (BioLegend), Allophycocyanin (APC) conjugated anti-CD11b/c (Novus Biologicals), APC conjugated anti-CD29 (Novus Biologicals), APC conjugated anti-CD90 (Novus Biologicals), APC conjugated anti-VEGFR2 (Novus Biologicals). The labelled ad-SVF was analyzed with FACSCalibur (BD Bioscience). As a negative control, an isotype-matched IgG was used for each antibody.

### Culture of ad-SVF and ad-SVF spheroids

For the ad-SVF monolayer, ad-SVF were cultured in Dulbecco’s Modified Eagle’s Medium (DMEM, Gibco) containing 10% fetal bovine serum (FBS), 1% penicillin and streptomycin at 37 °C with 5% CO2. To generate ad-SVF spheroids, ad-SVF were placed in 96-well ultra-low attachment plates (ULA, Corning), which are U-bottom plates. According to the previous study [21], ad-SVF were cultured at a seeding density of 1 × 10^5^ cells/well under the same conditions for 3 days. The spheroid morphology was observed daily using a fluorescence microscope (Olympus).

### Cell migration assay

Human umbilical endothelial cells (HUVEC) were plated on the upper chamber of a 24-well transwell with an 8-μm pore size polycarbonate membrane (Corning). Chambers below the filters were seeded with ad-SVF or ad-SVF spheroids. After 24 h culture, the cells on the lower surface were fixed with formaldehyde for 15 min, stained with 0.1% crystal violet, and observed in 5 nonoverlapping visual fields of each well using a microscope.

### Live/dead staining

Live/Dead cell imaging kit (Invitrogen) was performed to evaluate cell viability of ad-SVF or ad-SVF spheroids according to the manufacturer’s instructions. Briefly, Live/Dead working solution was added to cells after cell culture medium was exchanged by PBS. After incubating for 15 min at 25 °C, cells were observed under a fluorescence microscope (Olympus).

### Matrigel plug angiogenesis assay

Matrigel plug assay was performed to evaluate angiogenesis as previously described [22]. 2 × 10^5^ cells ad-SVF and equal amounts of ad-SVF spheroids were premixed with 500 μL Matrigel (1 mg/mL) at 4 °C. Then, the mixture was injected into the dorsal region of rat to generate Matrigel plug. After 2 weeks, the Matrigel plug was harvested and observed under a microscope by immunofluorescence.

### Tube formation assay

Pre-cooled μ-slide angiogenesis plate (Ibidi) was covered with 10 μL Matrigel (Corning) and incubated at 37 °C for 30 min to form a layer. HUVEC were suspended in serum-free endothelial cell growth medium (Sham), ad-SVF culture medium supernatant (ad-SVF), and ad-SVF spheroids culture medium supernatant (ad-SVF_sp_). Then, cells were seeded onto the former matrix at a density of 10,000 cells/well. After incubating at 37 °C for 6 h with 5% CO2 in a humidified atmosphere, all plates were observed by phase-contrast microscopy (Olympus).

### Enzyme-linked immunosorbent assay (ELISA)

Levels of basic Fibroblast Growth Factor (bFGF), Hepatocyte Growth Factor (HGF) and Vascular Endothelial Growth Factor A (VEGF-A) in medium supernatant obtained from ad-SVF or ad-SVF spheroids culture at 24, 48, 72 h after seeding were determined by ELISA kits (Elabscience) according to the manufacturer’s instructions. A microplate reader (Tacan) was used to detect the absorbance, and concentrations were calculated on the amount growth factor per 10^4^ cells at each time points.

### Cell labelling and tracking

Ad-SVF and ad-SVF spheroids were labelled with both the CellTracker™ CM-DiI and DiI-C_18_(5)-DS (Molecular Probes) before transplantation according to the manufacturer’s instructions. Briefly, CM-DiI (2 μg/mL) and DiI-C_18_(5)-DS (2 μg/mL) were performed to incubate cells for 5 min at 37 °C and following 15 min at 4 °C. Labelled ad-SVF or ad-SVF spheroids were injected to bladder and detected by IVIS Spectrum small-animal in vivo imaging system (PerkinElmer) and fluorescence microscope after 4 weeks.

### Experimental design and surgical procedures

According to previously described, PBOO model was firstly established in rats with some modifications [23]. Briefly, the rats were anesthetized with sodium pentobarbital (50 mg/kg) and placed on a thermostatic blanket at 37 °C. A midline incision was made in the lower abdomen to expose the bladder neck, and a 1.2 mm external diameter of needle was placed adjacent to the bladder neck. After ligation, the needle was removed and TGF-β (1 μg, 100 μL) was injected into the bladder wall.

After 4 weeks, all rats were randomly divided into three groups. The sham group: the bladder was exposed and the obstruction was resolved. Then, 100 μL phosphate buffer saline (PBS) was injected into the 4 places (25 μL/place) of bladder detrusor layer (the anterior, left, right, and posterior walls of bladder). The ad-SVF group: ad-SVF (2 × 10^6^ cells, 100 μL) was injected into same places of bladder wall after bladder exposed and obstruction resolved. The ad-SVF spheroids (ad-SVF_sp_) group: ad-SVF spheroids (total cells at 2 × 10^6^ cells, 100 μL) were transplanted into same places of bladder wall under the same condition. Polyethylene (PE)-50 tube was inserted into bladder dome in all rats for the following experiments.

### Bladder function analysis

The bladder functions of all rats in each group were evaluated by MedLab (Jenkintown, USA) at 4 weeks after the transplantation of ad-SVF or ad-SVF spheroids. Briefly, one end of the PE-50 tube was connected to the top of the bladder, and the other end was drawn under skin and fixed on the back skin surface before 3 days. Then, the PE-50 tube was connected to a pressure transducer and a venous pump using a 3-way stopcock, and then, physiologic saline (37 °C) was infused (0.5 mL/min). Continuous changes of intravesical pressure were recorded by MedLab software under conscious condition of rats. Bladder capacity (Maximum bladder capacity, MBC) was determined by the urination occurred, and post-void residual urine volume (PVR) was also measured.

### Histological and immunohistochemical staining

After urodynamic studies, all rats were sacrificed under complete anesthesia. After detecting by IVIS Spectrum small-animal in vivo imaging system, each of rat bladder was cut into two parts. One of parts was fixed with 4% paraformaldehyde solution, and another was cryopreserved in OCT compound (Tissue-Tek) for immunofluorescence staining. Paraffin sections were stained with hematoxylin and eosin (HE) for morphological evaluation and Masson for fibrotic assessment of the bladder tissues. Anti-CD31 antibody and anti-proliferating cell nuclear antigen (anti-PCNA) antibody were, respectively, used to measure the microvessel density and proliferation in bladder wall according to our previous protocol [24]. The terminal transferase–mediated deoxyuridine triphosphate nick-end-labelling (TUNEL) assay (Roche) was used to evaluate the apoptosis in bladder tissues depending on the manufacturer’s instructions. The positive cells were all analyzed in 5 random fields of each section.

### Immunofluorescence staining

The cryosections were fixed in acetone for 3 min and blocked with PBS + 5% bovine serum albumin (BSA, Biofroxx) for 30 min; then, primary antibody (CD31 or Ki67) was used overnight at 4 °C. A fluorescent secondary antibody Alexa Fluor 488 (Abcam) was performed on the sections, and nuclei was stained with 4,6-diamidino-2-phenylin-dole (DAPI, Beyotime). Subsequently, random 5 fields were observed to count CD31 (green) or Ki67 (Green) positive cells by fluorescence microscope (Olympus), respectively.

### Western blot analysis

A total protein extraction kit (Solarbio) was used to extract bladder tissues or cells proteins. The protein samples were subjected to sodium dodecyl sulfate (SDS)-polyacrylamide gel electrophoresis and transferred onto polyvinylidene difluoride membranes (EMD Millipore). The membranes were incubated with primary antibodies (bFGF (1:1000, Bioss), HGF (1:1000, Bioss), VEGF-A (1:1000, Bioss), AKT (1:2000, Proteintech), pAKT (1:2000, Proteintech), GSK-3β(1:1000, Proteintech), pGSK-3β(1:2000, Proteintech), and GAPDH (1:2000, Proteintech)) overnight at 4 °C after blocked with 5% non-fat milk, then followed by a secondary horseradish peroxidase-conjugated antibody (anti-rabbit or anti-mouse (1:5000, Cell Signaling)) for 2 h at room temperature. Enhanced chemiluminescence system (ECL kit) was used to detect the immunoblot signals, which were quantified by scanning densitometry using the ImageJ analysis system (NIH).

### Statistical analysis

All data were expressed as mean ± standard deviation (SD). Statistical analysis for comparisons of two groups was evaluated by independent sample *t* test, while for multiple groups, one-way analysis of variance was performed and the post hoc Tukey test was conducted the further comparison between groups. *P* < 0.05 was considered as a statistical signification.

## Results

### Characterization of ad-SVF and ad-SVF spheroids

**Fig. 1:**
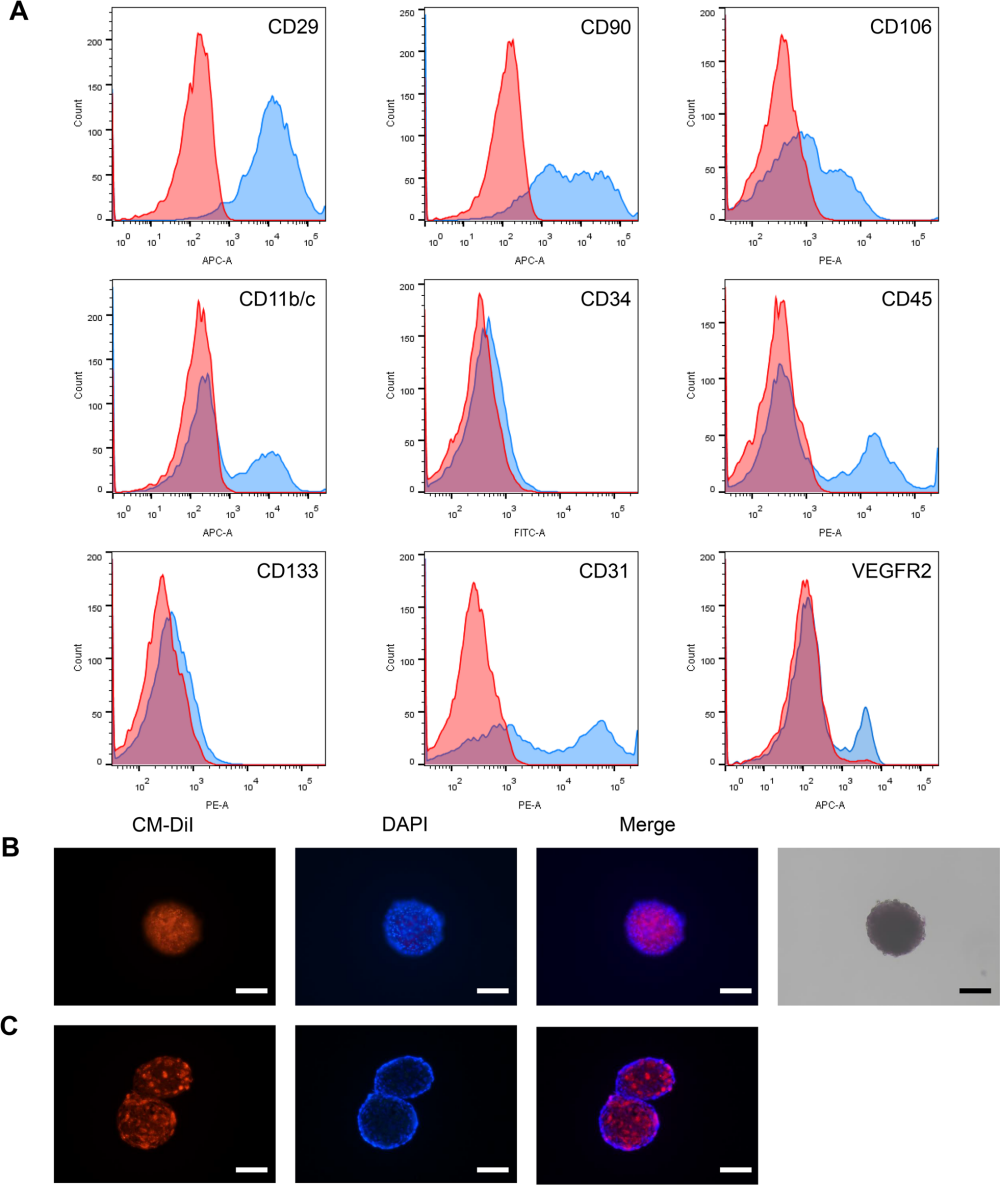
Characterization of ad-SVF and ad-SVF spheroids. **A** Representative flow cytometry histograms of ad-SVF. **B** Representative images of CM-DiI (red) labelled ad-SVF spheroids and nuclear marked DAPI (blue). Ad-SVF spheroids culture at day 3 (brightfield). **C** Representative images of CM-DiI labelled ad-SVF spheroids and nuclear marked DAPI (cross section). Scale bar = 100 μm

The results of flow cytometric analysis showed that the isolated ad-SVF is mainly composed of three different cells which expressed mesenchymal (CD29, CD90, CD106), hematopoietic (CD11b/c, CD34, CD45, CD133), and endothelial (CD31, CD34, VEGFR2) markers (Fig. 1A). After 3 days culture, the ad-SVF and ad-SVF spheroids were further analyzed using flow cytometric. There was not statistically significant between ad-SVF and ad-SVF spheroids. The diameter of most spheroids ranged from 100 to 300 μm (Fig. 1B). We observed that the inside of ad-SVF spheroids also has a mount of cells from the cross section, not as like a ping-pang ball (Fig. 1C).

### Enhanced secretion of angiogenic growth factors in ad-SVF spheroids culture

**Fig. 2:**
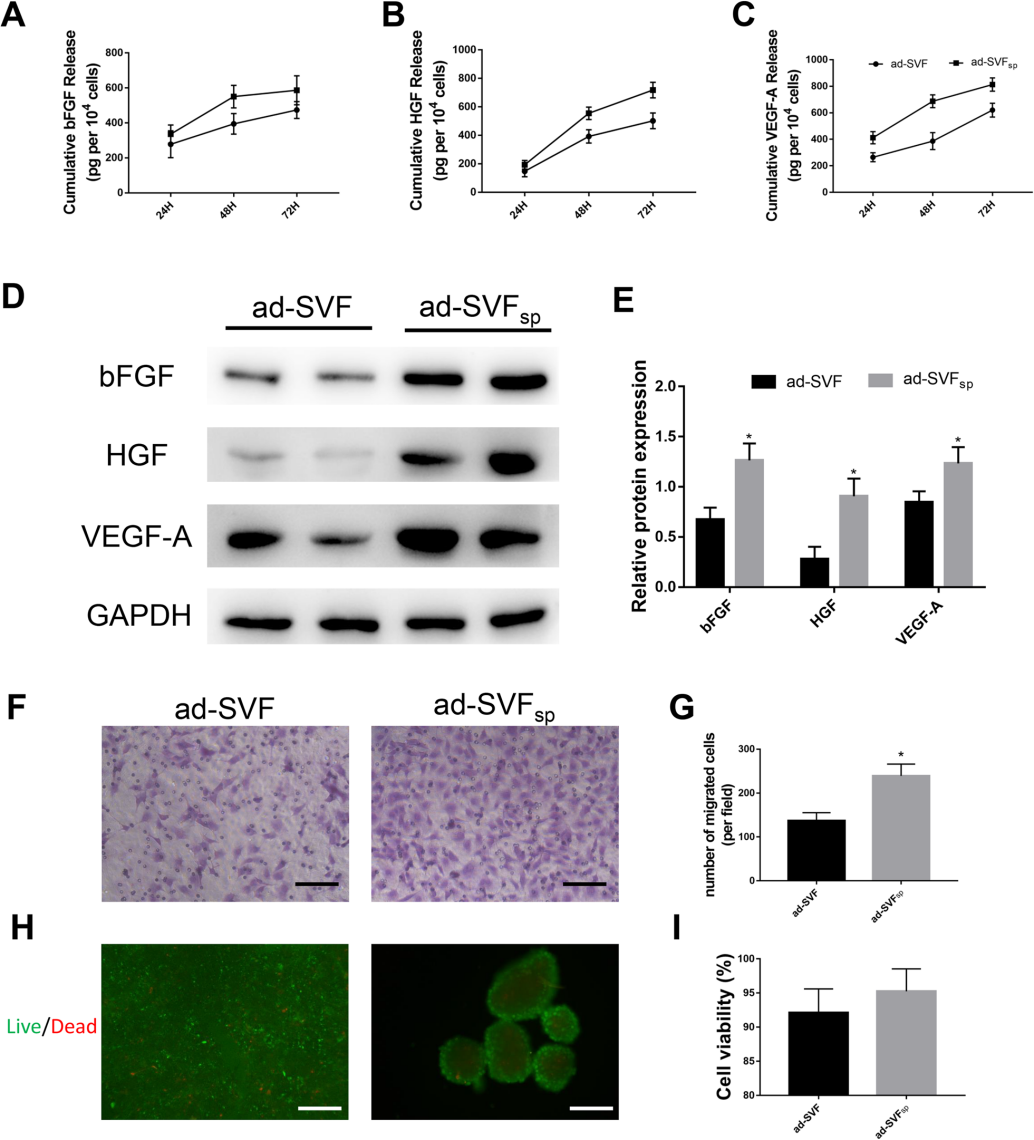
Enhanced expression of growth factors in ad-SVF spheroids. **A**–**C** Profiles for the cumulative release of bFGF, HGF, and VEGF-A from ad-SVF and ad-SVF spheroids as determined by ELISA at 24, 48, 72 h after seeding. **D**, **E** Relative abundance of bFGF/GAPDH, HGF/GAPDH, and VEGF-A/GAPDH were quantified according to western blots at day 3. **F** Representative images of migration effect on HUVEC. **G** Quantitative analysis of the mean number of migrated cells per field was performed. **H** Live/Dead fluorescence imaging of ad-SVF and ad-SVF spheroids. **I** Quantitative analysis of cell viability between ad-SVF and ad-SVF spheroids. Scale bar = 100 μm. *p < 0.05 (ad-SVF vs ad-SVFsp)

Compared with ad-SVF culture, ad-SVF spheroids can secret more angiogenic growth factors at 48 h and 72 h after seeding. We found that the levels of bFGF, HGF and VEGF-A in culture media are all gradually increasing in 3 days culture (Fig. 2A–C). We further detected the content of bFGF, HGF and VEGF-A in cells according to the western blot analysis and found that bFGF, HGF and VEGF-A are all significantly higher in ad-SVF spheroids than that in ad-SVF (Fig. 2D, E). A transwell migration assay was conducted to explore the effect of ad-SVF or ad-SVF spheroids on HUVEC. The number of migrated HUVEC was significantly increased in ad-SVF spheroids than that in ad-SVF (Fig. 2F, G). A Live/Dead imaging kit was performed to explore the cell viability between two groups. The results showed that cell viability was no significant statistic between two groups (Fig. 2H, I).

**Fig. 3:**
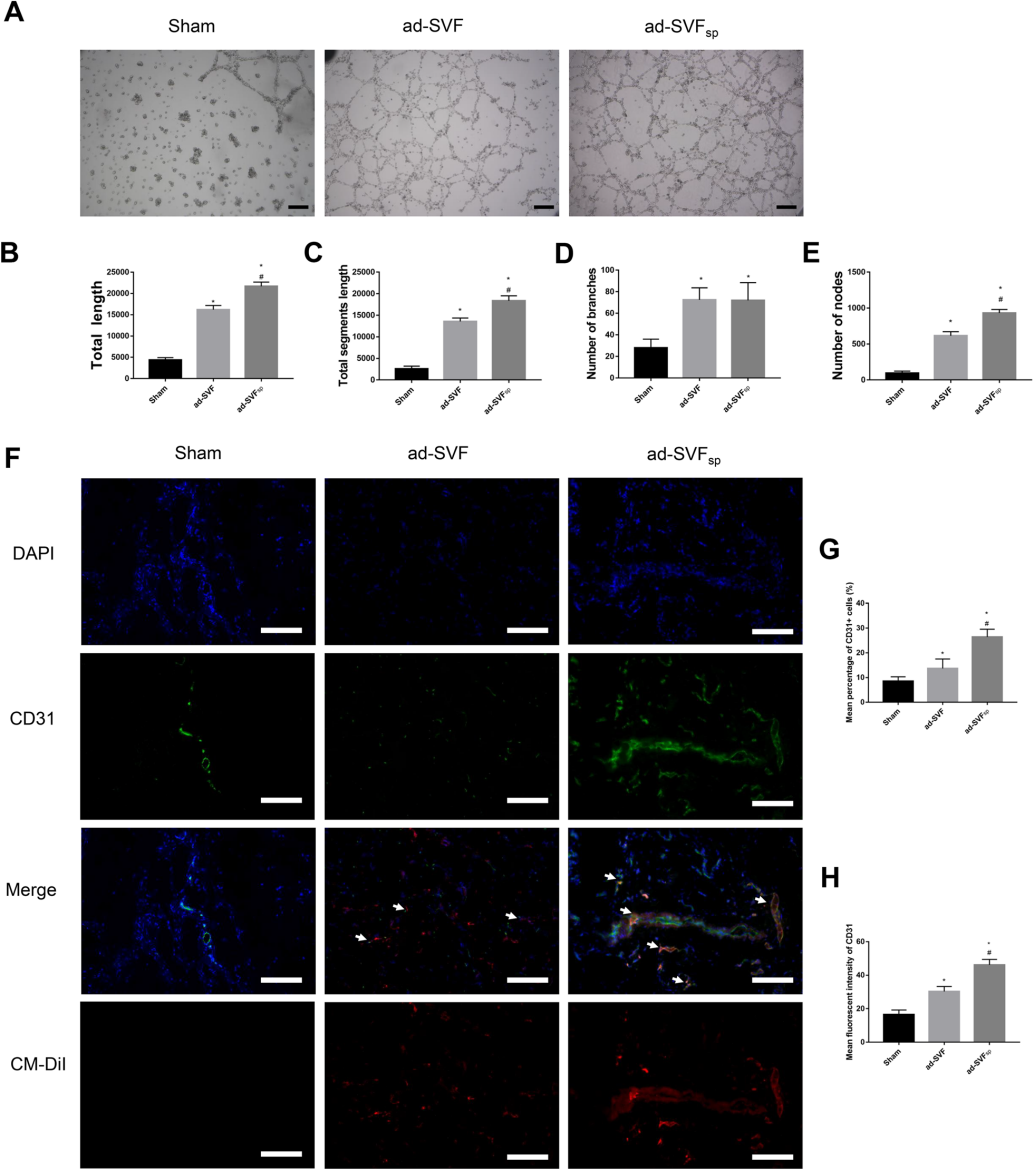
Ad-SVF spheroids promoted angiogenesis. **A** Representative images of tube formation assay under different culture medium. **B**–**E** Quantification of total length, total segment length, number of branches, and number of nodes were analyzed by ImageJ’s angiogenesis plugin. **F** Representative images of CM-DiI labelled cells (red), the expression of CD31 by immunofluorescence (green), and nuclear marked DAPI (blue) in Matrigel of each group (Arrows represent vessel). **G**, **H** Semi-quantitative analysis of the mean percentage of CD31 positive cells and the mean fluorescent intensity of CD31. Scale bar = 100 μm. *p < 0.05 (ad-SVF, ad-SVFsp vs Sham); ^#^*p* < 0.05 (ad-SVFsp vs ad-SVF)

Besides, we performed tube formation assay to explore the angiogenic potential of ad-SVF and ad-SVF spheroids-conditioned media. More tubules formed in the ad-SVFsp group than that in the ad-SVF group (Fig. 3A–E). Meanwhile, the matrigel plug angiogenesis assay showed that the angiogenic capability of ad-SVF or ad-SVF spheroids are stronger than the sham group and more capillaries can be observed in the ad-SVF_sp_ group than that in the ad-SVF group (Fig. 3F–H).

### Promoted the bladder function recovery by ad-SVF spheroids grafted

**Fig. 4:**
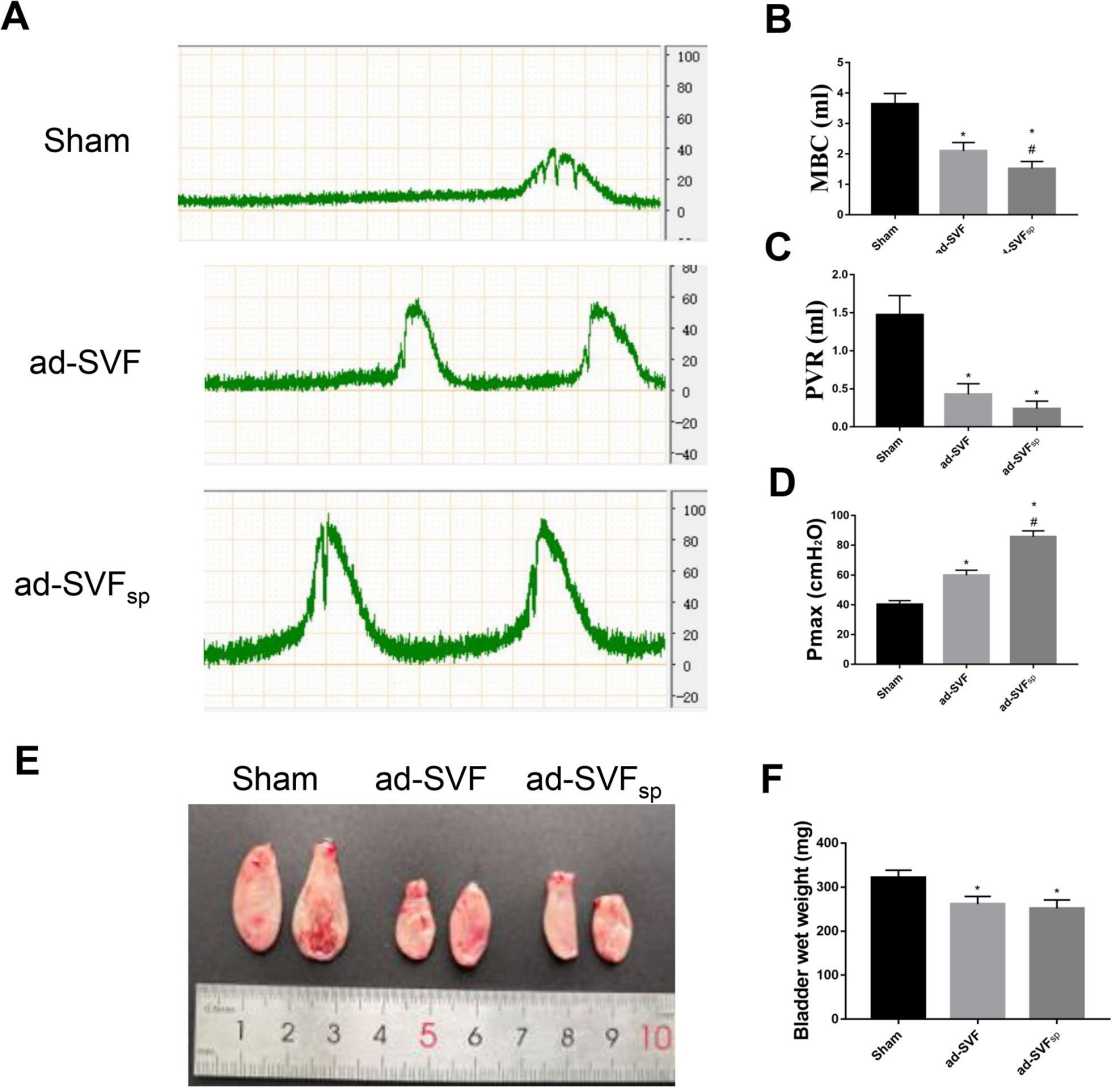
Ad-SVF spheroids improved bladder functions. **A** Representative images of urodynamics recording in each group (the peaks mean maximum pressure during bladder contraction). **B** Statistical chart of MBC in each group. **C** Statistical chart of PVR in each group. **D** Statistical chart of Pmax in each group. **E** Representative images of isolated bladder. **F** Statistical chart of bladder wet weight in each group. *p < 0.05 (ad-SVF, ad-SVFsp vs Sham); ^#^*p* < 0.05 (ad-SVFsp vs ad-SVF)

At 4 weeks after treatment, urodynamic studies were performed to determine whether ad-SVF or ad-SVF spheroids improved bladder function recovery. Representative cystometrograms (Fig. 4A–D) revealed that the bladder functions were significantly improved in the ad-SVF group and ad-SVF_sp_ group than that in the Sham group. Maximum bladder capacity (MBC) and post-void residual urine volume (PVR) were significant decrease in the ad-SVF group and ad-SVF_sp_ group, and maximum urination point pressure (Pmax) was elevation in the above two groups than that in the Sham group. Meanwhile, in the ad-SVF_sp_ group, there was a significant improvement in bladder functions. We also found that the wet weight of bladder in the Sham group was heavier than that in the Sham group and ad-SVF group, and there was no statistical significance between the ad-SVF group and the ad-SVF_sp_ group (Fig. 4E, F).

**Fig. 5:**
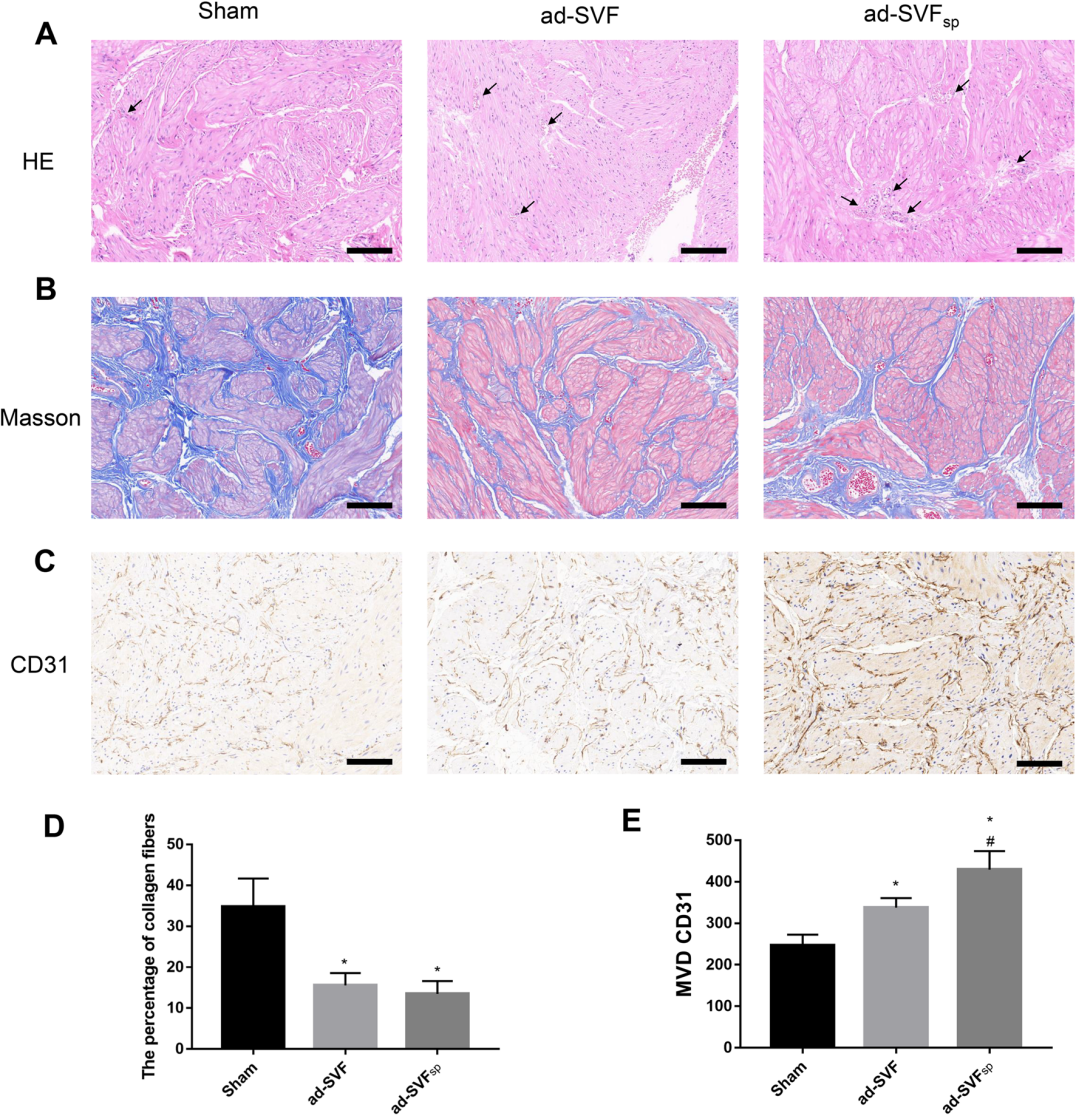
Ad-SVF spheroids attenuated the damage of underactive bladder. **A**–**C** Representative images of HE, Masson, and CD31 staining in each group (black arrows indicate small blood vessels). **D**, **E** Semi-quantitative analysis of the percentage of collagen fibers and MVD CD31 was performed by using Image J software. Scale bar = 100 μm. *p < 0.05 (ad-SVF, ad-SVFsp vs Sham); ^#^*p* < 0.05 (ad-SVFsp vs ad-SVF)

To investigate the structural changes of PBOO induced UAB, HE staining and Masson staining were performed at 4 weeks after transplantation. The bundles gap of smooth muscle was evident, and the fracture of muscle bundles could be obviously observed in the bladder induced by PBOO. Ad-SVF or ad-SVF spheroids treatment could attenuate these histological changes induced by PBOO (Fig. 5A). We also found that the deposition of collagen was decreased in the ad-SVF group and ad-SVF_sp_ group compared with that in the Sham group. The area ratio of collagen fibers was similar in the ad-SVF group and ad-SVF_sp_ group (Fig. 5B, D). HE staining also demonstrated that more blood vessels were presented in the ad-SVF group and ad-SVF_sp_ group (Fig. 5A). Furthermore, the results of immunohistochemical staining with CD31 showed that the microvessel density (MVD) significantly increased in the bladder after ad-SVF or ad-SVF spheroids treatment, and the MVD of bladder showed more in the ad-SVF_sp_ group than that in the ad-SVF group (Fig. 5C, E). Additionally, the immunofluorescence staining with CD31 was performed after cell tracking. The results showed that the vascular quantity increased in the ad-SVF group and ad-SVF_sp_ group than that in the Sham group. Additionally, the neovascularization around ad-SVF indicated that part blood vessels may be directly differentiated from ad-SVF (Fig. 7A–C).

### Increased cell retention rate by ad-SVF spheroids transplantation

**Fig. 6:**
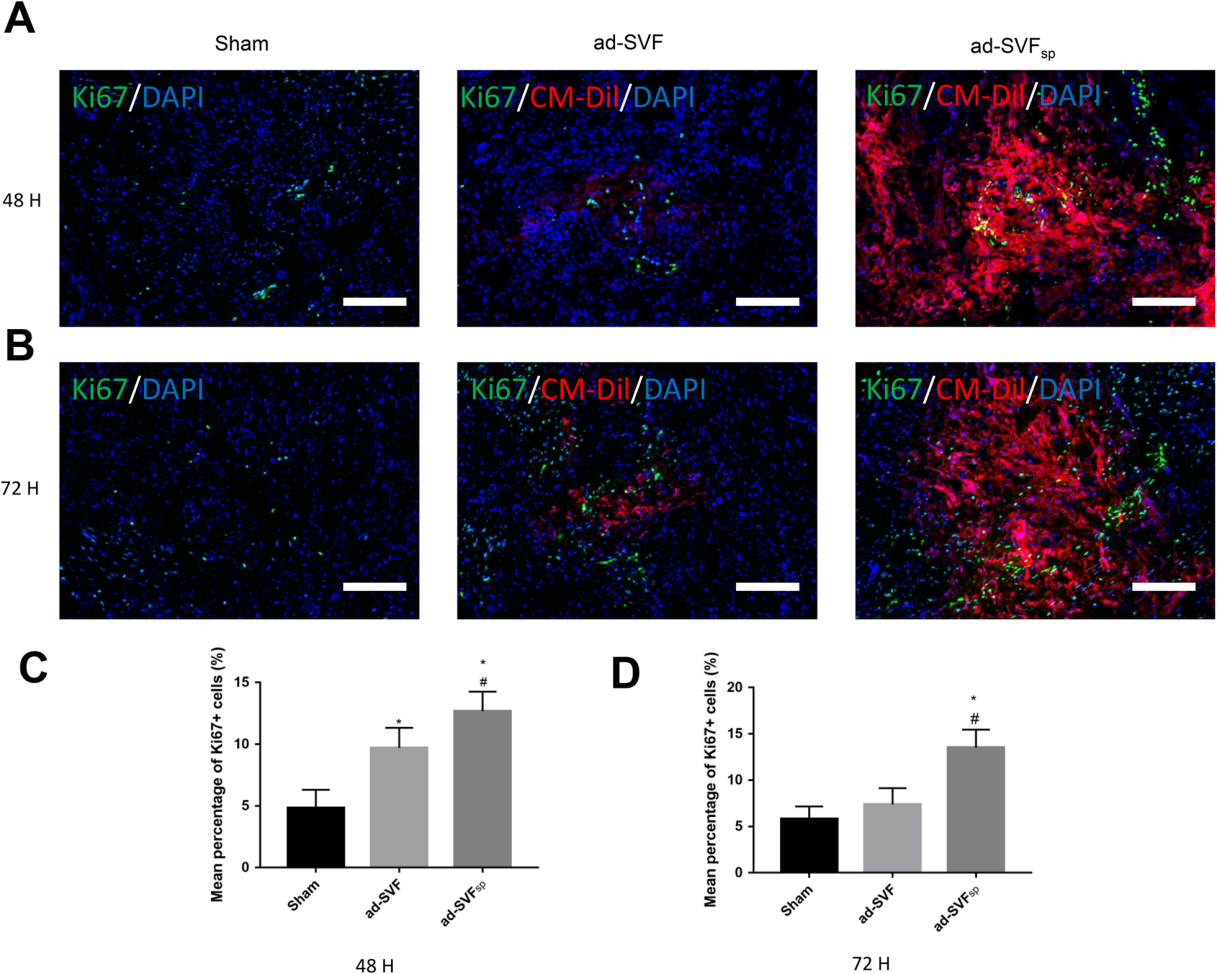
Ad-SVF spheroids promoted cell proliferation in underactive bladder. **A**, **B** Representative images of CM-DiI labelled cells (red), the expression of Ki67 by immunofluorescence (green), and nuclear marked DAPI (blue) in each group at 48 h and 72 h after cell transplantation. **C**, **D** Semi-quantitative analysis of the mean percentage of Ki67 positive cells at 48 h and 72 h after cell transplantation. Scale bar = 100 μm. *p < 0.05 (ad-SVF, ad-SVFsp vs Sham); ^#^*p* < 0.05 (ad-SVFsp vs ad-SVF)

**Fig. 7:**
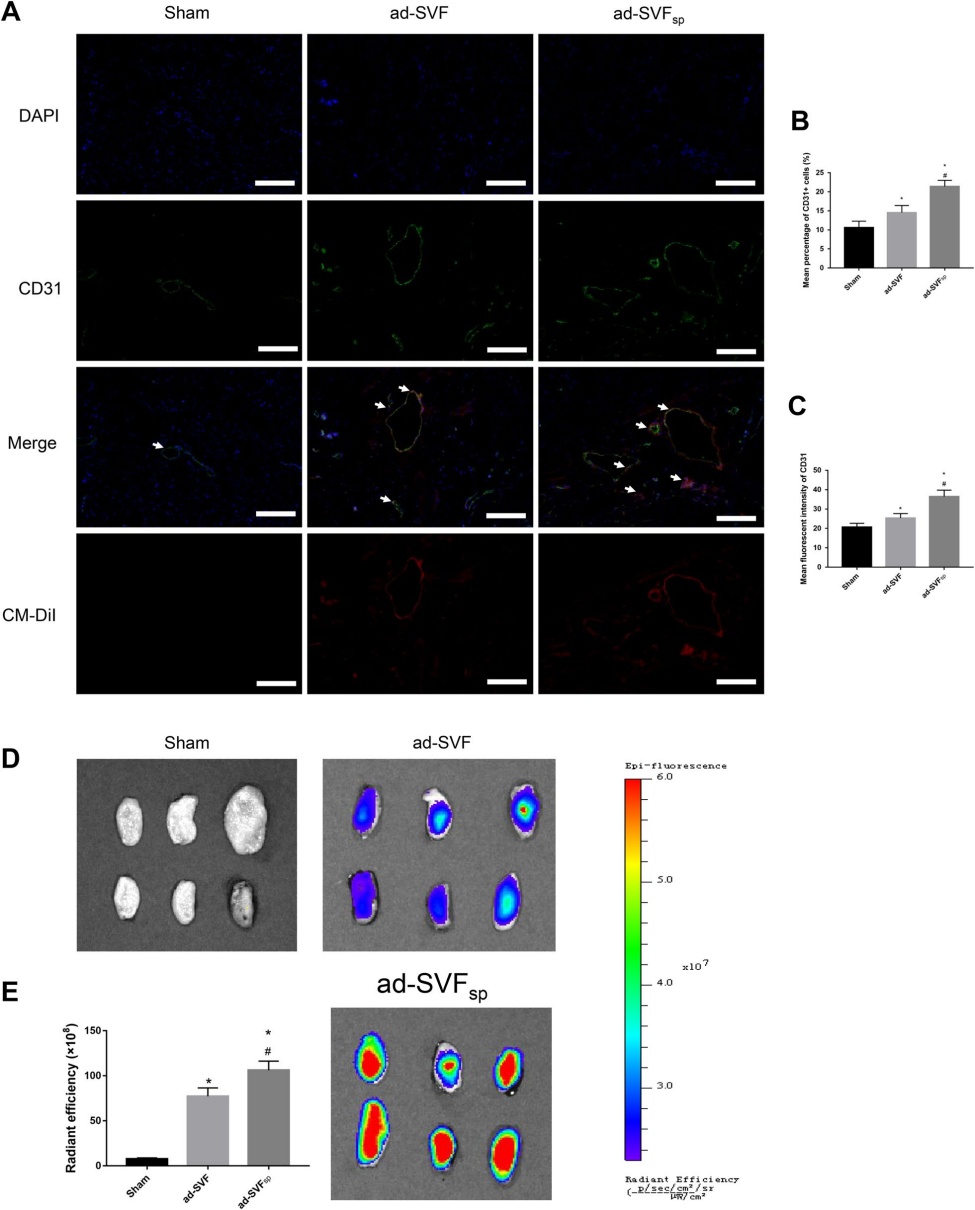
Cell tracking in rats treated with CM-Dil and Dil-C18(5)-DS labelled ad-SVF or ad-SVF spheroids at 4 weeks after cell transplantation. **A** Representative images of CM-DiI labelled cells (red), the expression of CD31 by immunofluorescence (green), and nuclear marked DAPI (blue) in each group. (Arrows represent vessel). **B**, **C** Semi-quantitative analysis of the mean percentage of CD31 positive cells and the mean fluorescent intensity of CD31. **D** Representative fluorescence images of bladders in vivo imaging system. **E** Semi-quantitative of CM-Dil and Dil-C18(5)-DS labelled cells in bladder of each group. *p < 0.05 (ad-SVF, ad-SVFsp vs Sham); ^#^*p* < 0.05 (ad-SVFsp vs ad-SVF)

To explore the early fate of cells, we selected two time points, 48 h and 72 h after cell transplantation, and observed the fate of cells in the bladder wall by immunofluorescence. We found that when same number of cells were injected, more cells of spheroids remained in the bladder wall. The results showed that ad-SVF spheroids have a higher retention rate and proliferation ability than ad-SVF (Fig. 6A–D). To explore cell retention rate after ad-SVF or ad-SVF spheroids treatment at 4 weeks after cell transplantation, the bladders were analyzed using an in vivo imaging system and fluorescence microscope. The results showed that more vessels were found in the ad-SVF_sp_ group and indicated that ad-SVF spheroids showed a longer-lasting therapeutic effect (Fig. 7A–C). Furthermore, the bladder fluorescence intensity could be observed in the ad-SVF group and ad-SVF_sp_ group, whereas the bladder fluorescence intensity of ad-SVF_sp_ group was significantly higher than that in the ad-SVF group, indicating that ad-SVF spheroids could stay in the bladder longer than ad-SVF (Fig. 7D, E). Observation of the CM-DiI labelled cells revealed the similar result by fluorescence microscope (Fig. 7A–C).

### Effects of ad-SVF spheroids on cell apoptosis and proliferation in bladder tissues

**Fig. 8:**
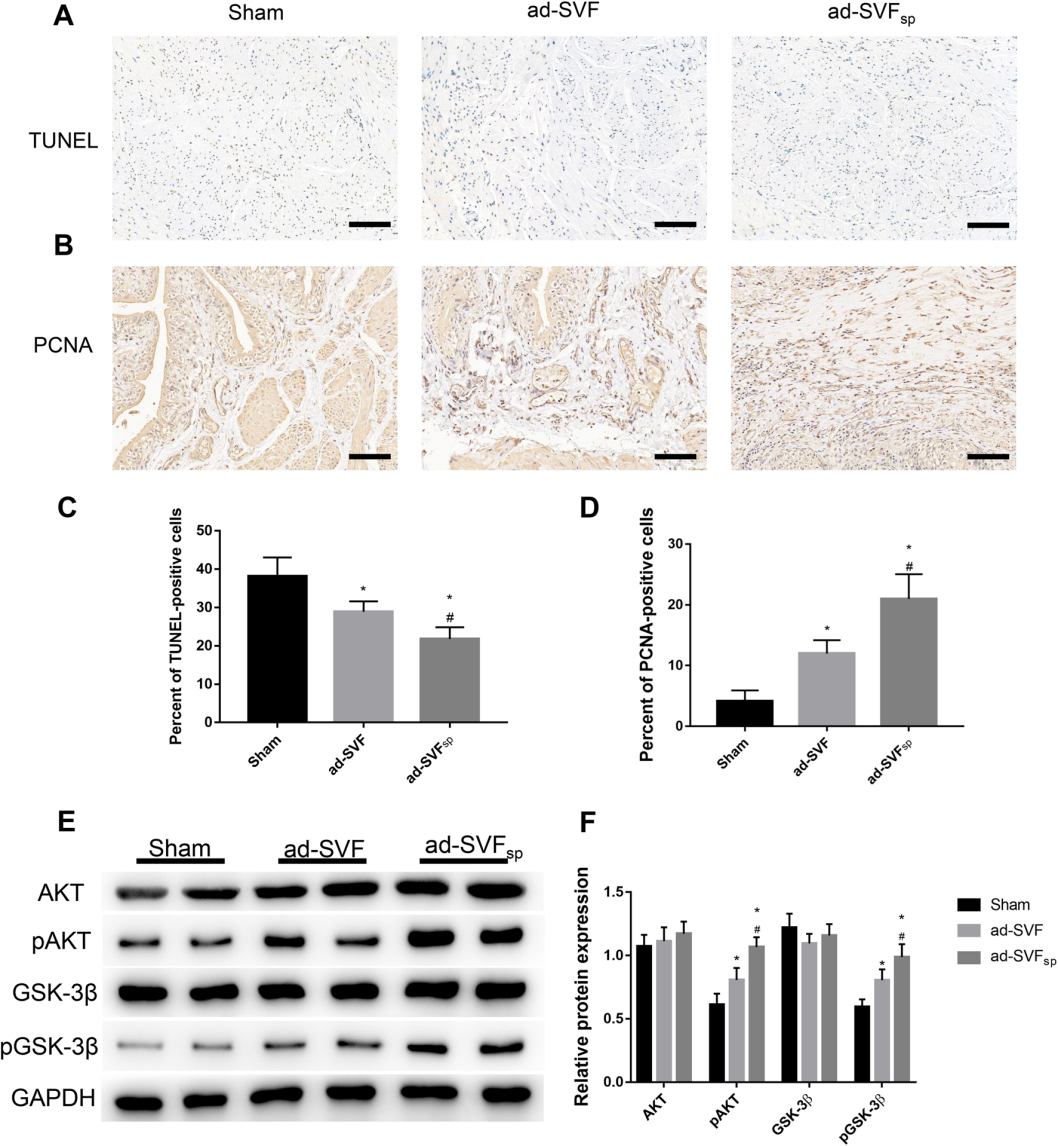
Effects of ad-SVF spheroids on cell apoptosis and proliferation. **A** Representative images of TUNEL staining in each group. **B** Representative images of PCNA staining in each group. **C**, **D** Semi-quantitative analysis of TUNEL-positive cells and PCNA-positive cells was performed by using Image J software. **E** Representative western blot images of AKT, pAKT, GSK-3β, pGSK-3β, and GAPDH. **F** Relative abundance of AKT/GAPDH, pAKT/GAPDH, GSK-3β/GAPDH and pGSK-3β/GAPDH were quantified according to western blots. Scale bar = 100 μm. *p < 0.05 (ad-SVF, ad-SVFsp vs Sham); ^#^*p* < 0.05 (ad-SVFsp vs ad-SVF)

The apoptosis and proliferation of cells in the bladder tissue from three groups were evaluated by the TUNEL staining and PCNA staining. The results of TUNEL staining showed that the percent of TUNEL-positive cells was decreased after ad-SVF or ad-SVF spheroids transplantation, and less TUNEL-positive cells were showed in the ad-SVF_sp_ group (Fig. 8A, C). Meanwhile, the results of PCNA staining exhibited that the percent of PCNA-positive cells was highest in ad-SVF_sp_ groups and higher in the ad-SVF group than that in the Sham group, indicating a high cell proliferation (Fig. 8B, D). Therefore, these results demonstrated that ad-SVF may provide therapeutic effects for UAB induced by PBOO and ad-SVF spheroids may have a better protective effect.

The present study detected the AKT/GSK-3β pathway by western blotting. Ad-SVF spheroids significantly increased the expression of phosphorylation AKT (pAKT) and subsequently increased the expression of phosphorylation GSK-3β (pGSK-3β) (Fig. 8E, F). These results indicated that ad-SVF spheroids effectively suppressed cell apoptosis and promoted cell proliferation via activation of the AKT/GSK-3β pathway in rats.

## Discussion

In present study, we first demonstrated that ad-SVF spheroids significantly improved bladder functions and attenuated bladder injury in UAB rats, and enhanced engraftment of ad-SVF. Meanwhile, we also found that the ad-SVF spheroids promoted angiogenesis in bladder tissues, which was involved in the paracrine of bFGF, HGF, and VEGF-A, and part endothelial progenitor cells in ad-SVF. Furthermore, the results also showed that the ad-SVF spheroids promoted cell proliferation and anti-apoptosis via the activation of AKT/GSK-3β pathway. Following clinical studies on the proof of the safety and effectiveness of ad-SVF transplantation for the functional recovery of damage organs [25–27], our study provides important clinical significance for the improvement of underactive bladder induced by partial bladder outlet obstruction.

Underactive bladder is common but under researched bladder disorder, which is known to the change of bladder morphology and function [10, 28]. Recently, studies are devoted to exploring the effective therapies to treat UAB, due to its complex and multifactorial etiology [29]. Study has reported that MSC could improve the structure and function of UAB [30]. In our study, bladder functions were evaluated by cystometry and the results showed that administration of ad-SVF spheroids was also able to attenuate the histological changes and improve bladder function, indicating the potential effect of ad-SVF spheroids on UAB. However, as contractility of bladder smooth muscle strips is also an important factor to evaluate functional recovery of UAB, it is necessary to detect the contractility of bladder smooth muscle strips in the future.

Ad-SVF provide a rich of stem cells, which consists of heterogeneous cell populations [12, 14, 31]. Many studies demonstrated that ad-SVF could promote tissue regeneration and organ function recovery [19, 25, 32]. Previous research demonstrated that adipose-derived regenerative cells transplantation improved bladder dysfunction and attenuated the changes of bladder histology [9]. Furthermore, in clinical trial, ad-SVF were confirmed as a safe and effective therapy [27, 33]. Therefore, in current study, ad-SVF spheroids were applied to attenuate histological damage, improve bladder function and stimulate bladder tissue regeneration. However, we only explored the early therapeutic effects of ad-SVF spheroids treatment, and the long-term protective effects of ad-SVF spheroids in UAB induced by PBOO also need to be verified.

Currently, various of stem cells are studied in bladder tissue recovery and regeneration, but the underlying mechanisms of cellular recovery and regeneration remain poorly understood. One possible mechanism is the secreted growth factors of implanted ad-SVF into bladder wall, which may play a crucial role in cell migration and proliferation. Research has investigated that ad-SVF could secrete various of growth factors including bFGF, HGF, VEGF-A, and so on [34]. In this study, ad-SVF spheroids could secrete more growth factors than ad-SVF and promote endothelial cell migration, which indicate a better recovery effect in UAB induced by PBOO. Meanwhile, according to the results of tube formation assay and matrigel plug angiogenesis assay, ad-SVF spheroids had more beneficial to form blood vessels in vitro and in vivo. bFGF, as a member of FGFs, is demonstrated to suppress cell apoptosis and promote proliferation [35]. HGF was reported to be a potential anti-fibrotic factor in damaged tissues and pro-angiogenic factor, which has similar effects with VEGF-A [36, 37]. In our experiments, UAB induced by PBOO exhibited serious bladder muscle bundles injury and bladder fibrosis. Ad-SVF spheroids administration attenuated the damage of bladder and intermuscular collagen accumulation. Meanwhile, the percent of TUNEL-positive cells reduced and the percent of PCNA-positive cells increased after ad-SVF spheroids transplantation. Additionally, we found that ad-SVF spheroids facilitated the formation of microvessels through immunohistological analysis. We also found that ad-SVF spheroids have an effect on cell proliferation in bladder tissue at 48 h and 72 h after cell transplantation.

Furthermore, AKT/GSK-3β pathway may act as a crucial role in cell apoptosis and proliferation. Previous researches proved that AKT plays an important role in the process of cell proliferation, migration, and angiogenesis [30, 38]. GSK-3β is also well documented to participate in a complex array of critical cellular processes, which was firstly founded in rat skeletal muscle as a serine/threonine kinase. This versatile protein is involved in numerous signaling pathways that influence metabolism, differentiation, migration, cell cycle progression and survival [39]. The increased pAKT and pGSK-3β usually lead to the improvement of cell proliferation and the inhibition of cell apoptosis [40]. bFGF has been reported to be related to cell survival by regulating downstream pathways, which involves in the increased expression of phosphorylating AKT and GSK-3β [30, 41]. Our results also showed that the phosphorylation of AKT and GSK-3β expression level both increased in the ad-SVF group and ad-SVF_sp_ group. These results indicated that ad-SVF, as a type of stem cell, also has a protective effect on cells and ad-SVF spheroids have better effects in proliferation, anti-apoptosis, and neovascularization. Other possible mechanism may relate to the direct differentiation of implanted stem cells. Several studies reported that stem cells could differentiate into smooth muscle and endothelial cells to promote the regeneration of bladder tissue [42, 43]. According to the immunofluorescence staining, we observed that part CM-DiI labelled ad-SVF differentiate into endothelial cells to promote blood supply recovery in bladder.

To the best of our knowledge, several strategies to improve the survival rate and transplantation rate of stem cells have been explored, including stem cell genetic engineering modification [30], tissue engineering scaffolds [13, 44], and stem cell combined with growth factor transplantation [45]. Transplanting ad-SVF as 3D spheroid may enhance cell survival and improve therapeutic effect in bladder. Compared with dissociated stem cells, spheroid could maintain more extracellular matrix (ECM) without enzymatic treatments. Our previous research demonstrated that ECM enhances the therapeutic effects of stem cells in ischemic tissue through its bioactive ingredients [46]. In addition, cells inside the spheroid are under mild hypoxia, which naturally preconditioned to transplant environment [47]. Therefore, in this study, we demonstrated that ad-SVF spheroids enhanced cell survival rate and retention rate in bladder after cell transplantation. However, the underlying mechanism of 3D spheroid needs more experimental proofs.

Although ad-SVF spheroids show great potential therapeutic effects in UAB, there are some limitations of this study. Firstly, the diversity of animal models indicates the complexity of exploring the etiology of UAB, like aging models [48], bladder outlet obstruction models [23], diabetic bladder dysfunction models [30], and neurogenic models [29]. Further researches need to prove whether ad-SVF spheroids are effective for various of UAB. In addition, only the short-term cell tracking used in this study due to the transient labelling of CM-DiI and DiI-C18(5)-DS. The long-acting labelling technique to track ad-SVF cell should be considered in the subsequent studies. At last, our data confirmed that ad-SVF spheroids could attenuate bladder injury and improve bladder functions attributing to the paracrine effect of ad-SVF spheroids. Further investigation on inhibiting the release growth factors from ad-SVF spheroids is needed to identify.

## Conclusion

In conclusion, the current study demonstrated that ad-SVF spheroids could ameliorate the bladder damage and improve the functions and symptoms of underactive bladder induced by PBOO in a rat model. The therapeutic effect of ad-SVF spheroids might be ascribed to the paracrine, differentiation, and high retention of ad-SVF spheroids, which suppressed cell apoptosis and promoted cell proliferation and neovascularization via AKT/GSK-3β pathway. These results suggest that the ad-SVF spheroids may be a potential treatment for UAB in the future.

## Data Availability

The datasets used and/or analyzed during the current study are available from the corresponding author on reasonable request.

## Abbreviations

UAB: Underactive bladder
ad-SVF: Adipose stromal vascular fraction
PBOO: Partial bladder outlet obstruction
DUA: Detrusor underactivity
LUTS: Lower urinary tract symptoms
ICS: International Continence Society
BPH: Benign prostatic hyperplasia
TGF-β: Transforming growth factor-β
MSC: Mesenchymal stem cells
PBS: Phosphate-buffered saline
FITC: Fluorescein Isothiocyanate
PE: Phycoerythrin
APC: Allophycocyanin
FBS: Fetal bovine serum
ULA: Ultra-low attachment plates
HUVEC: Human umbilical endothelial cells
bFGF: Basic Fibroblast Growth Factor
HGF: Hepatocyte Growth Factor
VEGF-A: Vascular Endothelial Growth Factor A
PVR: Post-void residual urine volume
HE: Hematoxylin and eosin
anti-PCNA: Anti-proliferating cell nuclear antigen
TUNEL: Terminal transferase–mediated deoxyuridine triphosphate nick-end-labelling
BSA: Bovine serum albumin
MBC: Maximum bladder capacity
Pmax: Maximum urination point pressure
MVD: Microvessel density
